# Stroke Patients’ Free-Time Activities and Spatial Preferences During Inpatient Recovery in Rehabilitation Centers

**DOI:** 10.1177/19375867221113054

**Published:** 2022-07-18

**Authors:** Maja Kevdzija, Ruzica Bozovic-Stamenovic, Gesine Marquardt

**Affiliations:** 1 Chair for Social and Health Care Buildings and Design, Faculty of Architecture, Technische Universität Dresden, Germany; 2 Department of Building Theory by Design, Institute of Architecture and Design, Faculty of Architecture and Planning, TU Wien, Vienna, Austria; 3 Department of Architecture, College of Design and Engineering, National University of Singapore, Singapore

**Keywords:** stroke rehabilitation, free-time activities, spatial preferences, rehabilitation environment, architectural layout, patient shadowing

## Abstract

**Objectives:**

To investigate which spaces stroke patients visit in their free time while undergoing inpatient recovery in rehabilitation centers, what activities they engage in, and what kind of spaces they want.

**Background:**

Research studies consistently show that stroke patients are highly inactive during rehabilitation. Much remains unknown about what patients do in their free time and how the built environment might affect their behavior and activities.

**Methods:**

Patients’ free-time activities were recorded via patient shadowing (*n* = 70, 840 hr), and their spatial preferences were collected using a survey (*n* = 60) in seven rehabilitation centers. Each participant was observed over one typical day (12 consecutive hours). Their activities, durations, and locations were recorded using floor plans and time log sheets.

**Results:**

Six main themes emerged from the analysis of shadowing data and patient surveys: (1) spending most free time in their room, (2) corridor as the overlooked activity hub, (3) food and beverage stations as triggers of activity, (4) wanting to socialize, (5) variety of common spaces for different activities is desired, and (6) common room’s atmosphere, comfort, style, and view are important. Even though socializing with other patients was mentioned as a primary reason for visiting common spaces in the survey, patients spent most of their free time alone.

**Conclusions:**

Corridor emerged as a space with great potential to motivate and support various activities of patients. Patients’ free-time activities could contribute to their recovery, and the built environment may play a role in facilitating and supporting these activities.

## Introduction

Stroke is a sudden and devastating event that causes particularly complex disability in adults compared to other conditions (Adamson et al., 2004). In addition to the typical motor impairments such as one-sided muscle weakness or paralysis, people after a stroke might experience cognitive impairments involving memory, orientation, language, and attention (Tatemichi et al., 1994), speech impairments (Flowers et al., 2016), and visual impairments (Rowe et al., 2009). These various disabilities can affect their independence (Desrosiers et al., 2002), psychological well-being (Brown et al., 2012), and quality of life (De Wit et al., 2017).

The numbers of people affected by stroke are expected to steadily increase in the following decades, resulting from the aging population, population growth, improved stroke care, generally longer life span, and increased main stroke risk factors (Feigin et al., 2017). This is especially evident in Europe, where the number of affected individuals is expected to rise by 27% between 2017 and 2047 (Wafa et al., 2020). The predicted increase in stroke numbers emphasizes the significance of improving stroke care in all of its parts, where the physical environment of rehabilitation settings may play a role in the care process.

### Specificities of Stroke Rehabilitation in Germany

About 25% of all patients after a stroke in Germany are discharged to an inpatient rehabilitation facility directly following acute treatment (Heuschmann et al., 2010). Returning to one’s prestroke activities and lifestyle is essential for well-being and an important goal during rehabilitation (Singam et al., 2015). For this to happen, neurological rehabilitation focuses on regaining mobility and independence in the activities of daily living in order to return home and to the previous workplace (Fertl, 2011).

Similar to the United States and many other European countries, rehabilitation of stroke patients in Germany takes place in hospitals or inpatient rehabilitation centers. What distinguishes Germany’s neurological rehabilitation system is the division of stroke patients into six rehabilitation phases, Phases A, B, C, D, E, and F, based on their Barthel index score, which determines their level of independence (Fertl, 2011). In the unstable acute A (and sometimes B) phase, the diagnostic and medical treatment occurs in stroke units or hospital wards (Heuschmann et al., 2010). Stroke patients recovering in rehabilitation centers usually belong to Phases B, C, and D. They are already conscious, cooperative, and at least partially mobile to participate in the offered treatment procedures (Fertl, 2011). Activating rehabilitation care, promoting mobility and independence, and providing targeted functional treatment are some of the aspects of rehabilitation for these patients (Wallesch, 2015).

Rehabilitation centers are, therefore, a particular type of healthcare facility for several reasons: (1) there are over 50 centers dedicated entirely to neurological rehabilitation in Germany (Rehakliniken.de, n.d.); (2) stroke inpatients’ stay in these facilities can last from several weeks (Nikolaus et al., 2006; Bussmann et al., 2018) to several months in individual cases; (3) they are transitional places to recover and regain the lost functions; and (4) most patients are independently mobile and required to attend therapies and meals on their own in different building areas outside of their wards. These characteristics distinguish a rehabilitation center as an environment where activity, mobility, and recovery speed are crucial; patients need to relearn the lost functions and prepare to return to their previous living environments (Nanninga et al., 2015).

### Importance of Activity in Rehabilitation

Early in rehabilitation, avoiding bed rest and being active (in scheduled therapy and free time) are crucial aspects that contribute to patients’ faster recovery, psychological health, and well-being (Winstein et al., 2016). It is important to begin exercising as soon as possible after a stroke to reduce the adverse effects of bed rest and inactivity, to take full advantage of the heightened neuroplasticity early following a stroke, and to initiate the important process of promoting self-efficacy and self-monitoring (Winstein et al., 2016). Thus, it is critical to prevent the poststroke cycle of decreased physical activity, which can lead to additional losses in functional capacity and an increased risk of secondary complications (Winstein et al., 2016). An entire spectrum of human movement could be recognized under the term “physical activity”: from the activities of daily living and recreational activities to exercise (Miles, 2007). Stroke patients view performing activities that make them get out of bed as a form of physical exercise, while reading or listening to the radio are seen as cognitive stimulation (Costa et al., 2021).

Even though activity is essential during rehabilitation, research studies conducted in different types of rehabilitation environments show that stroke patients spend a small portion of their day in scheduled therapy (Anåker et al., 2018; Åstrand et al., 2016; Blennerhassett et al., 2018; Chen et al., 2020; West & Bernhardt, 2012). In these studies, patients were found to have substantial free time between scheduled therapies and meals, mostly spent alone in their rooms. Patients also spent a large portion of their time in sedentary behavior (Barrett et al., 2018; Sjöholm et al., 2014). Their low activity levels during rehabilitation could be affected by different factors. Because patients can have a wide range of poststroke impairments and attend several various therapies each day, their mobility issues, pain, and general fatigue could reduce their wish or ability for additional free-time activities (Cowdell & Garrett, 2003; Cumming et al., 2016; Harrison & Field, 2015). Furthermore, according to the rehabilitation staff, institutional priorities, staff culture, and attitude can also be barriers to stroke patient activity during rehabilitation (Janssen et al., 2022). Patients report that the disempowering staff attitudes, inflexible rules, and a lack of opportunities to make choices contribute to their dependency (Luker et al., 2015), which could further limit their activity levels.

The built environment of rehabilitation facilities might also contribute to patients’ inactivity. While in rehabilitation centers, stroke inpatients’ rehabilitation is organized according to the prescribed therapy plan and consists of various therapy types, most commonly physiotherapy and occupational therapy (Hempler et al., 2018). A typical patient’s day includes scheduled therapies, meals, and free time between and after therapies. Even though they have a substantial amount of free time, patients report feelings of boredom (Cowdell & Garrett, 2003) and wish for more recreational and social activities (Luker et al., 2015). Patients also suggest that recreation in their free time is important since it relieves boredom and aids in stroke recovery (Luker et al., 2015). Physical environments of rehabilitation can hinder patients’ activity by creating barriers (Kevdzija & Marquardt, 2018), promoting loneliness and inactivity by not offering suitable and motivating common spaces (Anåker et al., 2019), and lacking opportunities for activities and additional exercise (Eng et al., 2014). The built environment of rehabilitation may also contribute to boredom by providing limited access to communal and outdoor spaces and the equipment for self-initiated therapy (Kenah, 2018). Still, much remains unknown about the ways the built environment might affect stroke patients’ behavior and activities, as this research field is still in its early stages (Lipson-Smith et al., 2021).

Stroke patients’ activity intensity, frequency, and duration during rehabilitation have been frequently researched (Fini et al., 2017). However, their free-time activities and the spaces they visit in their free time are still insufficiently explored and understood. Because patients do not spend a large portion of their day in active scheduled therapy, it is critical to examine and understand what they do in their free time and how their activity could be promoted outside of therapy time. Having access to common rooms with books, games, and computers, for example, might be a necessity for recovery after a stroke since it can create opportunities for activities and social interactions (Anåker et al., 2019; Janssen et al., 2014). The effect of environmental enrichment in the form of providing various materials (reading material, computer games, board games, puzzles, and music) in communal areas was examined for its effect on patients’ activities in several recent studies with mixed results (Janssen et al., 2014, 2021; Rosbergen et al., 2017). One of the main conclusions was that besides providing these materials, the activity-promoting culture in the rehabilitation facility needs to be created, and the built environment needs to be altered to improve stroke patients’ activity (Janssen et al., 2021). The materials provided in the communal spaces might be insufficient to motivate patient activity if these spaces are located far away and not visible from patient rooms (Anåker et al., 2017; Kevdzija & Marquardt, 2021). The spatial configuration of rehabilitation centers and the location and design of communal spaces outside of patients’ rooms could potentially support free-time activities; however, their contribution is not yet well understood. At the same time, little is known about the spatial preferences of patients.

***Because patients do not spend a large portion of their day in active scheduled therapy, it is critical to examine and understand what they do in their free time and how their activity could be promoted outside of therapy time***

Patients’ wishes and preferences are important to investigate, as they might influence their activity levels and participation during rehabilitation. Active involvement in setting rehabilitation goals and regaining autonomy during rehabilitation are important and can affect patients’ recovery motivation (Luker et al., 2015). Furthermore, patients stress that their motivation for rehabilitation is constantly changing and needs nurturing (Luker et al., 2015). In patients’ free time, when they are driving their own activity, nurturing motivation for activities and creating opportunities for independence may be essential for their recovery, not only by the treatment team but also via the built environment design.

Hence, it seems necessary to further examine the stroke patients’ spatial preferences and how the built environment could motivate, encourage, and support their free-time activities and recovery process. The large majority of previous research focusing on the rehabilitation environments for stroke patients had been conducted in acute rehabilitation and on a smaller scale of a stroke unit or a rehabilitation ward in a hospital. This study focuses on post-acute inpatient centers dedicated solely to rehabilitation. Patients undergoing recovery in these centers do not spend the entire day inside their ward. They are mobile and required to leave their ward (where their room is located) and attend therapies in different parts of the building several times per day, which widens the range of spaces they can visit within the whole building in their free time. This research study aims to provide insight into the activities and experiences of individual stroke inpatients during their free time in rehabilitation centers. It explores what patients do in their free time during rehabilitation, the types of spaces they visit, and their preferences regarding the spaces they wish for in rehabilitation centers.

## Method

This study uses a convergent parallel mixed methods research design (Creswell & Plano Clark, 2018) with the use of two research methods: patient shadowing (*n* = 70, a total of 840 hr) and patient survey (*n* = 60) to investigate patients’ free-time activities in seven rehabilitation centers. The purpose of collecting both shadowing (qualitative) and patient survey (qualitative and quantitative) data is to assess alternative viewpoints and gain a more profound understanding of the same phenomenon. The shadowing method involves “a researcher closely following a subject over a period of time to investigate what people actually do in the course of their everyday lives” (Quinlan, 2008, p. 1480). This method can be greatly adapted to the research setting and the research subject. For example, notation of positions on the floor plans can be added to accompany the field notes in studies where the role of the built environment is important. In contrast to behavioral mapping, the researcher using shadowing takes a nearly continuous set of field notes on the observation day (Mcdonald, 2005), combined with short, on-the-go interviews where participants explain or reflect on specific actions or behaviors (van der Weele & Bredewold, 2021). Shadowing, therefore, results in a “rich, dense and comprehensive data set,” which can then be analyzed as any other qualitative data (Mcdonald, 2005, p. 457). This differentiates shadowing from behavioral mapping (Ng, 2015), the method often used to investigate the time use of patients in healthcare settings.

In this study, we adopted an exploratory shadowing approach by combining descriptive observations with position and activity tracking on the floor plans and spontaneous on-the-go conversations (when possible) with the observed patients (Kevdzija, 2022). This paper focuses on the shadowing method’s observations and position and activity tracking aspects. One researcher (first author) shadowed patients in all public (entrance, lobby, café, and other areas accessible to all visitors) and semi-public (corridors and patients’ common areas) spaces of the centers throughout the whole day. Patient shadowing was chosen to record patients’ visited spaces, activities, and the duration of these activities in the built environment of rehabilitation centers using the floor plans and time log sheets (see Supplemental Material 1 for an example of data collection instruments in one of the centers). Each activity and interaction with space were recorded in the form of a position on the floor plan and the time and description on the time log sheet. All visible activities were recorded; for example, it could not be determined by observing if patients were performing a cognitive activity while sitting and doing nothing else. The activity was defined as any change of condition that could be observed. Certain activities were grouped; for example, if a patient was eating, not every movement was recorded—from taking a fork and a knife to cutting and eating, but the whole activity was recorded as “eating.”

To better understand the patients’ daily experiences in the centers, patients who were shadowed also completed a survey about their spatial preferences. Not all 70 observed patients were able (due to poststroke impairments and inability to write) or willing to fill out the survey, resulting in 60 completed surveys. The survey responses were used to supplement the shadowing data by identifying patients’ opinions and attitudes regarding common spaces at centers and their spatial preferences.

### Ethical Approval

This study was approved by the Ethical Committee at the Technische Universität Dresden (approval number: EK 452102016). Each patient who participated in the research study gave their consent, written or verbal (in the presence of a witness). The participants were able to drop out of the study at any time.

### Settings

The study was conducted in seven neurological rehabilitation centers in five different federal states in Germany. The centers were comparable in size and number of beds (see Supplemental Material 2 for the characteristics of participating centers).

The selected rehabilitation centers had different spatial configurations and different types and distribution of communal spaces, both inside (Supplemental Material 3) and outside the wards. All centers had a cafeteria on the ground floor, and some had additional common spaces outside the wards, such as an additional café, library, or winter garden. In the participating centers, meals were not a group activity. Every patient group had a time window when they could go to the main cafeteria or the dining room on the ward (depending on their mobility level) to eat. They could eat at any time within that time window.

### Participants

The participants were 70 stroke patients staying as inpatients in rehabilitation centers (Table 1). The medical staff selected 10 patients per center, considering their health status, psychological state, and the provided inclusion and exclusion criteria. The study included patients who: suffered a stroke, were able to move independently in the center (with or without the use of a wheelchair or a walker) and gave their consent for the study. The exclusion criteria were dementia, severe communication, and cognitive impairments, severe multi-morbidity (somatic, psychiatric, or psycho/geriatric), significant mobility impairment before stroke, and/or orthopedic, neurological, or other condition of consequence for the study. Patients were accommodated in different wards within the rehabilitation centers and were mobile enough to independently attend therapies and meals in the main cafeteria (see Supplemental Material 5 for all patient paths observed in one of the centers).

**Table 1.** Participants’ Characteristics.

Characteristic	Participants (*n* = 70)
Age	≥60
Gender	
Female	32 (45.7%)
Male	38 (54.3%)
Barthel index for mobility	
5 (wheelchair independent, including corners)	11 (15.7%)
10 (walks with help of one person)	16 (22.9%)
15 (independent, may use an aid, e.g., stick)	43 (61.4%)
Length of stay to time of observation (days)	Median: 19.5
Range	3–139
Patients in single rooms	58
Patients in double rooms	12

### Procedure

Data collection took place from September 2016 to May 2018. The potential study participants were approached by a staff member who was well known to them and the researcher (first author), usually during their free time in their room. After a brief explanation of the study and the research methods used, patients were asked to participate. If the patient agreed, they received a large print information sheet and a consent form. The observation day was also scheduled on that occasion. The researcher was given the patient’s therapy plan for the day to have insight into their scheduled activities. The participating patients were observed (shadowed) for 12 consecutive hours each (from 07:00 to 19:00 hr) on an ordinary working day in the center. The observation time amounted to 840 hr in all participating centers.

Each observed patient also completed a paper survey when this was possible due to limitations from a stroke. Patients were given the survey on the day of the observation and could complete it on that day or one of the following days. The survey consisted of 15 large print questions addressing different aspects of the built environment, such as the physical barriers patients experienced and spaces/places they liked to visit in their free time. This study focuses on the six questions investigating where patients go in their free time during rehabilitation (Table 2).

**Table 2.** Survey Questions.

Q1	Is there a place/space outside your room where you like to spend time during your free time? *(yes / no question)*
Q2	Which place/space do you visit? *(open-ended question)*
Q3	Why do you like this place/space? *(multiple choice question + other)*
Q4	How often do you visit this place/space? *(multiple choice question + other)*
Q5	Do you go there alone or with other patients/visitors? *(multiple choice question + other)*
Q6	What kind of space (room or place) would you like to have in the clinic? *(open-ended question)*

### Data Processing and Analysis

Shadowing data recorded in paper form were first digitalized. This data set contained paths on the floor plans, times and durations of activities, and their descriptions. Because all activity durations and locations were recorded, and because of the extensive duration of observations, it was possible to quantify some of the shadowing data. The absolute time spent in each area was calculated by aggregating the periods spent by the patient in each location. All of the time patients had between scheduled therapy, diagnostic appointments, and meals was considered free time. Therefore, all activities that were not scheduled were considered free-time activities.

All free-time activities were linked to their respective locations and counted for the number of observed events. The analysis focused on the locations within the rehabilitation centers’ buildings and the frequency of their visits. The determinant of the company was then added to each activity (was the patient alone, with another patient, with visitors, etc.) based on the symbols on the floor plans and the activity descriptions on the time log sheets. These accompanying notes where each activity was textually described were used to add the final layer: the type of activity.

The survey responses were analyzed separately from the shadowing data and then compared to identify how they diverged or confirmed the findings. Multiple-choice survey responses were analyzed for the prevalence of each response. The open-ended responses (Q2 and Q6) were translated from German and coded using NVivo Version 11 software. The analysis of Q2 responses consisted of counting the frequency of each mentioned space, which was then compared with the shadowing results in a joint display figure. For Q6, inductive thematic analysis was used to identify the prevalent themes in patients’ spatial preferences and opinions (see Supplemental Material 4 for the responses to Question 6), and the patients’ quotes were used to discuss and compare the shadowing and survey findings. The quantitative and qualitative results are presented in a narrative discussion structured into themes that emerged from the shadowing and survey data analysis.

## Results

Six main themes emerged from the analysis of shadowing data and patient surveys describing their free-time activities and locations: (1) spending most free time in their room, (2) corridor as the overlooked activity hub, (3) food and beverage stations as triggers of activity, (4) wanting to socialize, (5) variety of common spaces for different activities is desired, and (6) common room’s atmosphere, comfort, style, and view are important. These themes are presented below and discussed using shadowing data and patients’ survey responses.

### Spending Most Free Time in Their Room

**Figure 1.**
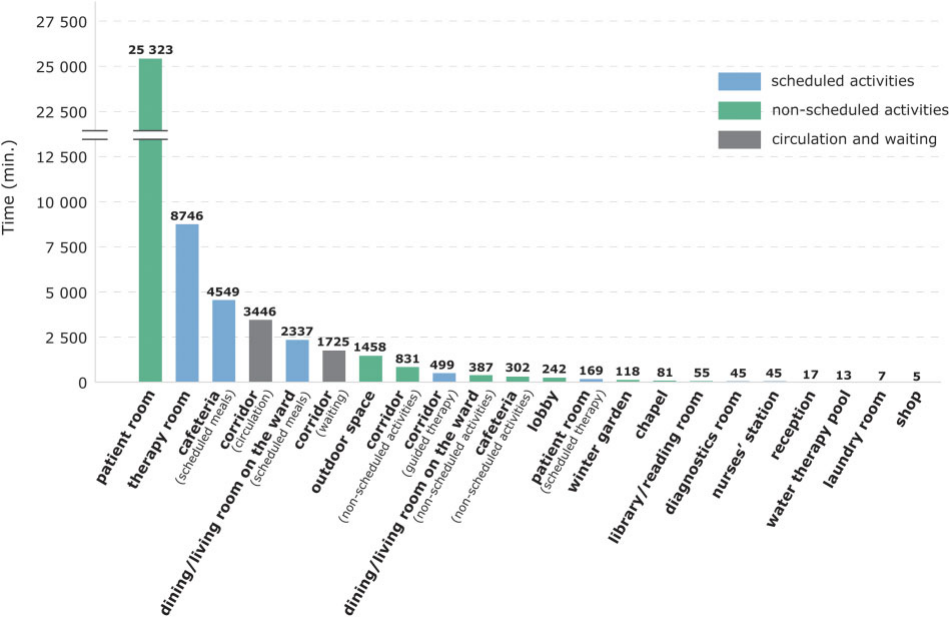
Absolute time and locations of patients’ scheduled and nonscheduled activities in seven rehabilitation centers.

Patients spent one-third (Mdn = 33%, IQR = 28.5%–37.3%) of the observation time in scheduled activities such as therapy and meals (16,407 of 50,400 min). Around 50% of the observation time (*Mdn* = 51.1%, IQR = 44.5%–57.3%) was spent inside their rooms (Figure 1). Because patients usually closed their doors, it is unclear if they remained inactive for the whole time in their rooms. Much of the patients’ time (*Mdn* = 10.28%, IQR = 7.64%–13.02%) was also spent in circulation and waiting for therapy in the corridors (Figure 1). The most common location of nonscheduled free-time activities was the outdoor area of the centers, and the main observed activities were smoking, sitting on a bench, and walking. On several occasions, a visitor would come and take the patient outside of the center’s building and stay away for a longer time (between 1 and 2 hr). It is unclear whether they spent this time outside or went to a café or another similar place in the area. The corridor was the indoor space where patients spent most of their free time compared to other formal common spaces such as the dining/living room on the ward, cafeteria, or lobby (Figure 1).

Around a third of all observed patients (*n* = 27) did not visit any common space inside the rehabilitation center’s building for non-scheduled activities, and 42 (60%) patients did not visit any outdoor spaces (Table 3). Indoor spaces were more frequently visited than outdoor spaces (Table 3), even though more time was spent outdoors (Figure 1). Most of them visited only one or two different spaces in their free time on the observation day (Table 3). Patients did not leave their rooms frequently, except for several smokers who went to the outdoor space multiple times (Table 3). Other patients mostly left their room once or twice during their free time.

**Table 3.** Frequency of Visited Locations in Patients’ Free-Time.

Description		Frequency	
		Indoors *n* (%)	Outdoors *n* (%)
Variety of visited spaces during the observation day	Zero visited spaces	27 (38.6%)	42 (60%)
	One visited space	25 (36%)	26 (37.2%)
	Two different visited spaces	17 (24%)	2 (2.8%)
	Three different visited spaces	1 (1.4%)	0
Number of times that a patient visited a space outside of their room in their free time during the observation day	Once	18 (25.7%)	13 (18.6%)
	Twice	15 (21.5%)	7 (10%)
	Three times	3 (4.3%)	3 (4.3%)
	Four times	4 (5.7%)	1 (1.4%)
	Five times	2 (2.8%)	0
	Six times	1 (1.4%)	1 (1.4%)
	Seven times	0	2 (2.8%)
	Eight times	0	1 (1.4%)

Even though many patients were observed to spend much of free time in their rooms, the majority of them indicated that there was a space/place outside of their room that they visited in their free time (Table 4, Q1) and that they visited this place every day or multiple times per week (Table 4, Q4).

**Table 4.** Survey Responses.

Question		Possible Choices	Number of Responses
Q1	Is there a place/space outside your room where you like to spend time during your free time? *(yes/no question)* *one response possible* (*n* = 59)	Yes	40
		No	19
	*Questions from Q2–Q5 were answered by patients who replied with “Yes” to Q1*		
Q2	Which place/space do you visit? *(open-ended question)* (*n* = 38)	Responses shown in Figure 2	
Q3	Why do you like this place/space? *(multiple choice question + other)* *multiple responses possible* (*n* = 40)	I can meet other patients there	18
		The view is interesting/beautiful	14
		It is large	8
		I can be undisturbed there	7
		It is close to my room	3
		I can do specific activities there	3
		It is small	2
		Other reasons (added by respondents): It is the only room to sit comfortably. It is bright for reading. For recovery and strengthening the muscles. The atmosphere is not clinical. I can observe people. There is coffee and snacks.	1 1 1 1 1 1
Q4	How often do you visit this place/space? *(multiple choice question + other)* *one response possible* (*n* = 40)	Every day	21
		Multiple times per week	13
		Once per week	5
		Other: When the weather is nice	1
Q5	Do you go there alone or with other patients/visitors? (multiple choice question + other) *multiple responses possible* (*n* = 40)	With visitors	19
		Alone	16
		With other patients	13
		Other	0
Q6	What kind of space (room or place) would you like to have in the clinic? *(open-ended question)* (*n* = 26)	Responses discussed throughout the Results section (also see Supplemental Material 4)	

### Corridor as the Overlooked Activity Hub

**Figure 2.**
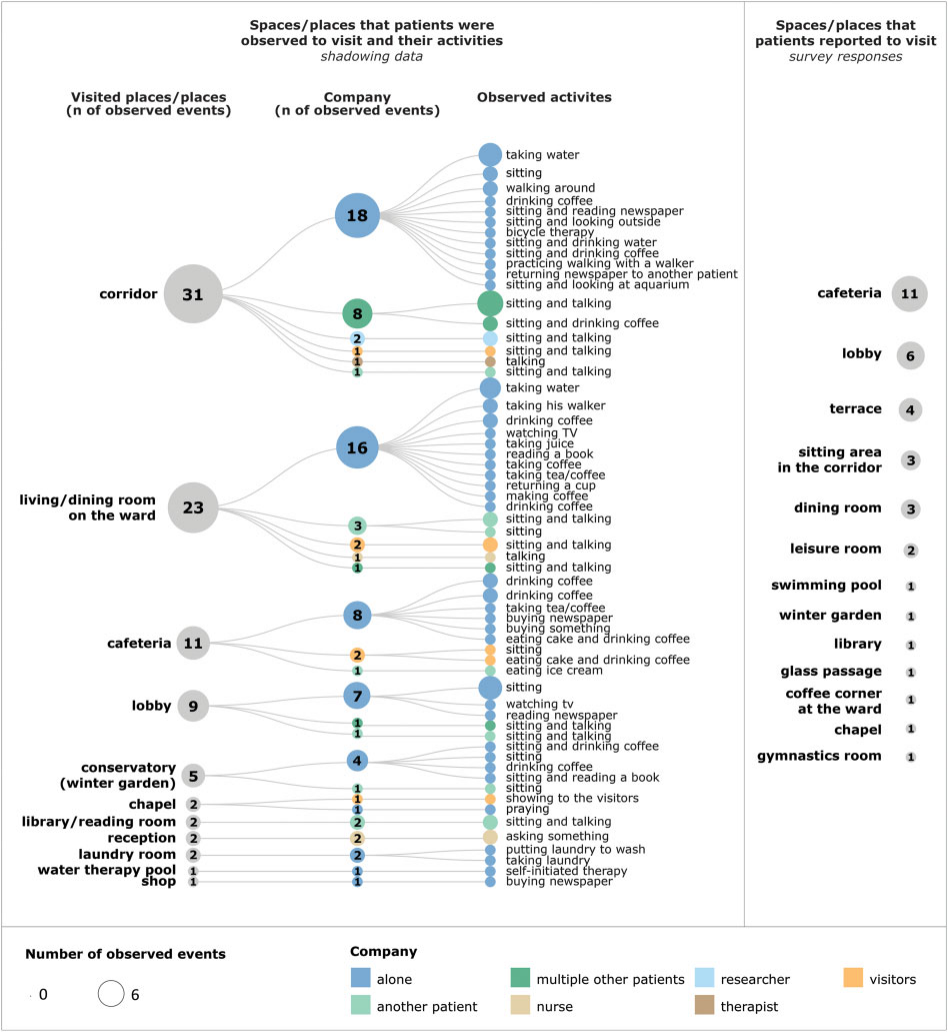
Patients’ observed free-time activities and their locations (left) and survey responses to Q2 (right).

Corridors and open seating areas in the corridors emerged as the most frequently visited spaces in patients’ free time for activities other than circulation (Figure 2). Formal communal spaces, such as the living/dining room on the ward and the main cafeteria, were less frequently visited by patients than the informal sitting areas in the corridor. Patients used corridors for a multitude of activities alone and with other patients. These activities included both sedentary behaviors, such as sitting and looking outside or sitting and reading a newspaper, and more physical activities, including walking around, practicing walking with a walker, or bicycle therapy (on an exercise bicycle provided in the corridor). Sedentary activities were observed more often (Figure 2). It was surprising that only three patients mentioned the sitting area in the corridor as a place they visited in their free time (Figure 2). The cafeteria and lobby were the most commonly reported spaces for spending free time.

### Food and Beverage Stations as Triggers of Activity

Many of the observed activities were related to sedentary behavior or connected to food and beverages (Figure 2). There were locations on the wards in most centers where patients could take a snack, water bottle, or make a coffee/tea. These food/beverage stations were placed inside the living/dining rooms on the ward or placed in the corridor or other common area. Even though the activities related to food and beverages were dominant in the patient observations, many of them were brief, such as taking water or coffee, and the patient would immediately return to their room. A different situation was observed in one of the centers with a winter garden (enclosed glass space with places to sit and views of the outdoor garden). Patients could buy coffee next to the winter garden, and they would usually stay to have the coffee here instead of returning to their room.

Only one patient mentioned “coffee and snacks” as a reason to visit a common space outside of their room (Table 4, Q3), and another patient stated that a “coffee corner at the ward” was the space they visited in their free time (Figure 2).

### Wanting to Socialize

Patients valued socializing with other patients. This was the most commonly reported reason why they liked visiting spaces other than their room during free time (Table 4, Q3). Additionally, most patients mentioned being in the company of others (visitors and other patients) when they visited their favorite space in the center (Table 4, Q5). One patient described the type of common room that he would like to have in his center as: “A room where you can sit with several people to read, talk, play. Close to my room on the same floor.” The cafeteria was also seen as an important place “to receive visitors, to meet with other patients.” Another patient expressed a wish for “a café or bistro open until 22:00 hr for socializing,” as the cafeteria in the center usually closed at 17:00 hr for visitors.

Even though many patients highlighted socializing as one of the main reasons to leave their rooms, they were observed to be alone in most free-time activities during their observation day (Figure 2). Furthermore, 16 patients (23%) stated that they visited their favorite space alone (Table 4, Q5). The corridor and library/reading room were the only spaces where a substantial number of observed activities were performed in the presence of one or more patients (Figure 2). These activities were sitting and talking or having a beverage together.

### Variety of Common Spaces for Different Activities Is Desired

Patients shared their ideas for common rooms where particular activities would be supported and which did not exist in many rehabilitation centers. Some wished for more offers related to music and entertainment, such as a music room, dancing room, singing room, cinema room, and TV room. One patient mentioned “a space where you can play board games” and another a “game room (for chess, etc.).” A “craft room” was also one of the spaces mentioned. One patient wished for “A relax room. Many sun-loungers in a botanical garden. Like indoor Central Park….” Two patients also described spaces where they would like to be alone: “a common area on my ward to sit, read” and “a smaller room where you can retreat.” Being undisturbed in a common space was reported by seven patients as a reason why they like a particular space outside of their room (Table 4, Q3).

### Common Room’s Atmosphere, Comfort, Style, and View Are Important

Fourteen patients (20%) mentioned the beautiful view as to why they visited a particular space outside their room (Table 4, Q3). Individual patients also reported the nonclinical atmosphere and the possibility to sit comfortably as reasons for visiting their favorite space in the center (Table 4, Q3). They described the comfort and style of their ideal common room as “a comfortable sitting room, homely furnished, not in the hospital-style, but as a café” and “a living room on the ward, comfortably furnished.” It was also suggested that the distance from the patient’s room was important. One patient describes wanting “A common room that is easy to reach. The common room here in the center is far away.” This patient also discusses the atmosphere of the existing common room in the center: “The room is very unpleasant, and it is not inviting to stay.” Having a bright and quiet space and a space without many people were also the qualities that some patients wanted in their ideal common room.

## Discussion

The amount of time that patients spent in their rooms observed in this study is consistent with all previous research conducted in various rehabilitation environments from the 1980s (Keith, 1980; Keith & Cowell, 1987) to recent years (Blennerhassett et al., 2018; Sjöholm et al., 2014). This is especially surprising in German postacute rehabilitation centers where patients are mainly mobile and not limited to their ward environment.

Corridors were the most commonly visited spaces for free-time activities during patient shadowing. In the study of De Wit et al. (2005) conducted in four European rehabilitation centers, patients in German and Swiss centers spent substantial time in the corridor, but their activities were not specified. Other studies on a rehabilitation ward or stroke unit level also show that patients spend a considerable amount of time in the corridors without specifying what the activities were or whether they were scheduled or not (Blennerhassett et al., 2018; Chen et al., 2020; Sjöholm et al., 2014). In this study, different free-time activities such as socializing, exercising, enjoying the view, walking around, and others were observed in the corridors separately from circulation, waiting, and therapy. This observed multifunctionality of corridors broadens the findings of previous studies investigating corridors in various healthcare environments. Corridors were identified as “spillover spaces” in a study conducted in spinal injury and brain injury rehabilitation wards and were used for therapy and exercise or as a storage area (Colley et al., 2018). Corridors were also recognized as frequent locations of everyday staff conversations (Long et al., 2007; González-Martínez et al., 2016). In our study, corridors were identified as important centers of patients’ free-time activities, but patients did not mention them as a common space they visited in their free time. The reason might be that corridors were not considered “common rooms/spaces” in the same way as, for example, a living room on the ward or a library. Corridors emerged as an informal common space that was not assigned to a specific activity but could be used in various ways and provided numerous corners and points of interest (e.g., sitting area, coffee machine, aquarium, therapy bicycle). Compared to the usual single living/dining room on the ward, often far away from most patient rooms, the corridor is there as soon as the patient steps out of their room. The patient can immediately see what is happening and decide to join the other patients’ activities or visit the favorite space alone or with visitors. Based on the variety of activities observed in the corridors, these spaces show great potential to be designed to encourage and support patients’ diverse free-time activities by providing various attractive features—seating configurations, food/beverage stations, interesting views, and exercise opportunities.

***Based on the variety of activities observed in the corridors, these spaces show great potential to be designed to encourage and support patients’ diverse free-time activities***

Many of the activities observed in the centers were related to food/beverages. While most patients in this study reported spaces connected to food and beverages (cafeteria, dining room, and coffee corner at the ward) as their favorite spaces/places to visit, only one patient mentioned “coffee and snacks” as the reason to visit this space. Patients stated other reasons, such as meeting others, view, size of the space, and distance from patients’ rooms, which all need to be considered during the design process to increase the attractiveness of common spaces. As observed in the winter garden case at one of the centers, strategically placing food and beverage stations near attractive common spaces may increase the likelihood of patients staying in this space. Alternatively, instead of staying in that common space, patients might return to their room while still exercising their mobility, which could benefit their recovery. Therefore, this study’s findings suggest that the location of food and beverage stations in combination with attractive common spaces in the rehabilitation center/patient wards is important. This could be used as one of the design strategies to potentially increase the mobility and activity of patients.

***strategically placing food and beverage stations near attractive common spaces may increase the likelihood of patients staying in this space.***

Stroke patients expressed a desire for more recreational activities in previous qualitative studies of their experiences in rehabilitation, such as access to reading materials, games, exercise equipment, and crafts (Luker et al., 2015), which was also reflected in the survey responses in this study. Additionally, patients reported visiting their favorite common space with other patients and visitors. They also mentioned meeting other patients as the main reason to visit a common space in their free time. This social aspect was also identified as important in a recent study in a stroke unit. In this research, stroke patients wished for a communal space to meet other patients, which could help reduce loneliness (Anåker et al., 2019). The wish to be with others has implications for designing communal spaces that enable patients to meet other patients and spend time with visitors outside their rooms. Providing more opportunities for patients to socialize might reduce boredom during rehabilitation and encourage participation in activities during their free time, which could positively contribute to their recovery (Kenah et al., 2022).

***The wish to be with others has implications for designing communal spaces that enable patients to meet other patients and spend time with visitors outside their rooms.***

Patients’ survey responses also indicate that a certain space is associated with the activity that could take place there. As patients give importance to the activities that could be performed in spaces they visit, particular care needs to be taken when planning for the activities that the common spaces would support and facilitate. Patients in this study expressed a desire for spaces that did not exist in their rehabilitation centers, ranging from entertainment rooms to spaces for relaxation and isolation, highlighting the current lack of offer in the participating centers.

Because patients’ spatial preferences are highly individual and their needs might change over time, the main challenge in designing common spaces in rehabilitation environments will be to provide flexibility and variety in terms of size and location, spatial qualities, and activities they enable. Another issue that should be considered when planning common spaces is the implementation of strategies to control the spread of the COVID-19 virus and other viral infections in common spaces. This might influence the particular design aspects such as the size of spaces, the use of materials, and ensuring the possibility of natural ventilation and the motivation of patients to leave their rooms and meet others.

According to the findings of this study, the common spaces currently available in rehabilitation centers might not offer enough variety and activity opportunities. Supporting patients’ free-time activities by providing diverse options to leave their rooms and autonomously engage in activities alone or with others could be essential for their recovery process. The built environment of rehabilitation centers should be recognized as an active component in the rehabilitation of stroke patients (Shannon et al., 2019). This study confirms the findings of the recent literature review on the built environments for inpatient stroke rehabilitation which identified multiple studies suggesting that attractive and accessible communal areas are important for the activity and well-being of stroke patients (Lipson-Smith et al., 2021). Patients’ skills practiced during their free-time activities might be especially important to prepare them for returning home in addition to the structured and supported activities in therapy sessions. Therefore, the built environment should offer opportunities for patients to participate in activities, meet others, and exercise their independence in their free time. Autonomy in free-time activities should be fostered not only in the built environment but also in the organizational culture of the rehabilitation facility (Janssen et al., 2021).

***Supporting patients’ free-time activities by providing diverse options to leave their rooms and autonomously engage in activities alone or with others could be essential for their recovery process.***

### Strengths and Limitations

The main strengths of this study are (1) each patient was accompanied for one whole day to investigate their everyday life and (2) daily activities of stroke patients from seven rehabilitation centers were analyzed in-depth with the use of (3) two complementing research methods providing two different perspectives on the phenomenon investigated. Since it involves close and extended observation of single individuals, shadowing patients in healthcare facilities can be challenging, and patients undergoing recovery might not be willing to participate in research studies spanning many hours. These challenges might have limited the use of shadowing in the patient population, and this study uses patient shadowing on such a large scale for the first time. Continuous patient observation over one typical day was proven valuable for studying how patients spend their days and should be considered in future research studies. A “series of snapshots of what individuals do at a given time” (Costa et al., 2021, p. 2) typical for research in healthcare environments likely cannot provide a complete picture of patients’ free-time behaviors and activities.

When interpreting the results, some limitations should be considered. Patients were only observed during working days, and their activity levels might have differed during weekends when they did not have scheduled activities. Younger stroke patients and those with severe communication impairments were not included in the study, and these two large patient groups might have different needs and experiences of free time. Patients’ behaviors might also have been altered due to close observations over the whole day. Other limitations are the sample size of 10 patients per center since preferences for common spaces are personal for each patient, the unavailability of survey responses for all participating patients, and the difficulty of controlling all the environmental variables that might have influenced patients’ activity. Even though the sample size might limit the potential generalizability of the results, this study provides a unique insight into stroke patients’ free-time activities that contribute to the understanding of their spatial needs during rehabilitation. The field research for this study was conducted pre-COVID-19, and the influence of the pandemic on everyday life in rehabilitation centers could not be observed.

## Conclusion

By exploring patients’ free-time activities in rehabilitation, this study adds to the growing body of research investigating the impact of the built environment on stroke patients’ activity levels. What patients do in their free time might contribute to their recovery, and the built environment could be one of the keys to enabling and supporting their activities. Rehabilitation centers are often primarily designed to fulfill the functions regarding the care processes; the communal spaces and the quality of their built environment are often neglected. The findings of this study suggest that the built environment shows the potential to support a wide range of patient activities. The design of these spaces should enrich patients’ free time and offer them opportunities for solitude, withdrawal, socializing, and performing various other activities. The insights into patients’ free-time activities and spatial preferences in this study could be used to reevaluate communal spaces in existing centers or inform future facilities’ design. Future research may look deeper into patients’ free time, their feelings, and preferences toward communal spaces and how the built environment could support their autonomy in free-time activities. Understanding patients’ spatial preferences is essential for designing built environments that enable them to engage as active participants in their rehabilitation.

## Implications for Practice

- Corridors were identified as informal communal spaces where a range of free-time activities occurred other than circulation, waiting, and guided therapy. When designing rehabilitation centers, special attention should be given to corridors, both on the wards and throughout the whole building, to provide varied areas and points of interest for patients’ free-time activities.
- Patients expressed a desire to socialize with other patients and to have a space outside of their rooms to receive visitors. One of the primary goals of communal space design should be to provide diverse environments and opportunities for patients to socialize with other patients and visitors.
- Because patients have different personalities and interests, they might differ considerably in communal spaces they find attractive. The communal spaces should provide environments for a variety of free-time activities, ranging from diverse entertainment options (cinema room, TV room) to spaces for physical and cognitive activity (dancing, crafting, and board games) and retreat and relaxation.
- Many of the observed free-time activities were associated with food and beverages. Strategically placed food and beverage stations, combined with attractive communal spaces, are likely to encourage patients’ activity in their free time, thereby promoting their mobility and recovery.

## Supplemental Material

Supplemental Material, sj-pdf-1-her-10.1177_19375867221113054 - Stroke Patients’ Free-Time Activities and Spatial Preferences During Inpatient Recovery in Rehabilitation Centers Click here for additional data file. Supplemental Material, sj-pdf-1-her-10.1177_19375867221113054 for Stroke Patients’ Free-Time Activities and Spatial Preferences During Inpatient Recovery in Rehabilitation Centers by Maja Kevdzija, Ruzica Bozovic-Stamenovic and Gesine Marquardt in HERD: Health Environments Research & Design Journal

Supplemental Material, sj-pdf-2-her-10.1177_19375867221113054 - Stroke Patients’ Free-Time Activities and Spatial Preferences During Inpatient Recovery in Rehabilitation Centers Click here for additional data file. Supplemental Material, sj-pdf-2-her-10.1177_19375867221113054 for Stroke Patients’ Free-Time Activities and Spatial Preferences During Inpatient Recovery in Rehabilitation Centers by Maja Kevdzija, Ruzica Bozovic-Stamenovic and Gesine Marquardt in HERD: Health Environments Research & Design Journal

Supplemental Material, sj-pdf-3-her-10.1177_19375867221113054 - Stroke Patients’ Free-Time Activities and Spatial Preferences During Inpatient Recovery in Rehabilitation Centers Click here for additional data file. Supplemental Material, sj-pdf-3-her-10.1177_19375867221113054 for Stroke Patients’ Free-Time Activities and Spatial Preferences During Inpatient Recovery in Rehabilitation Centers by Maja Kevdzija, Ruzica Bozovic-Stamenovic and Gesine Marquardt in HERD: Health Environments Research & Design Journal

Supplemental Material, sj-pdf-4-her-10.1177_19375867221113054 - Stroke Patients’ Free-Time Activities and Spatial Preferences During Inpatient Recovery in Rehabilitation Centers Click here for additional data file. Supplemental Material, sj-pdf-4-her-10.1177_19375867221113054 for Stroke Patients’ Free-Time Activities and Spatial Preferences During Inpatient Recovery in Rehabilitation Centers by Maja Kevdzija, Ruzica Bozovic-Stamenovic and Gesine Marquardt in HERD: Health Environments Research & Design Journal

Supplemental Material, sj-pdf-5-her-10.1177_19375867221113054 - Stroke Patients’ Free-Time Activities and Spatial Preferences During Inpatient Recovery in Rehabilitation Centers Click here for additional data file. Supplemental Material, sj-pdf-5-her-10.1177_19375867221113054 for Stroke Patients’ Free-Time Activities and Spatial Preferences During Inpatient Recovery in Rehabilitation Centers by Maja Kevdzija, Ruzica Bozovic-Stamenovic and Gesine Marquardt in HERD: Health Environments Research & Design Journal
