# Supplementary material for: Stroke Patients’ Free-Time Activities and Spatial Preferences During Inpatient Recovery in Rehabilitation Centers
Source: HERD. 2022 Jul 18;15(4):96–113. doi: 10.1177/19375867221113054 (PMC9523820; doi:10.1177/19375867221113054)
Supplement: Supplemental Material, sj-pdf-1-her-10.1177_19375867221113054 - Stroke Patients’ Free-Time Activities and Spatial Preferences During Inpatient Recovery in Rehabilitation Centers [file sj-pdf-1-her-10.1177_19375867221113054.pdf]

Observation sheet no. ______________ Date _________________

Patient no. _______________________

POS. NO.	TIME	REMARKS

Observation sheet no. ______________ Date _________________

Patient no. _______________________

EG

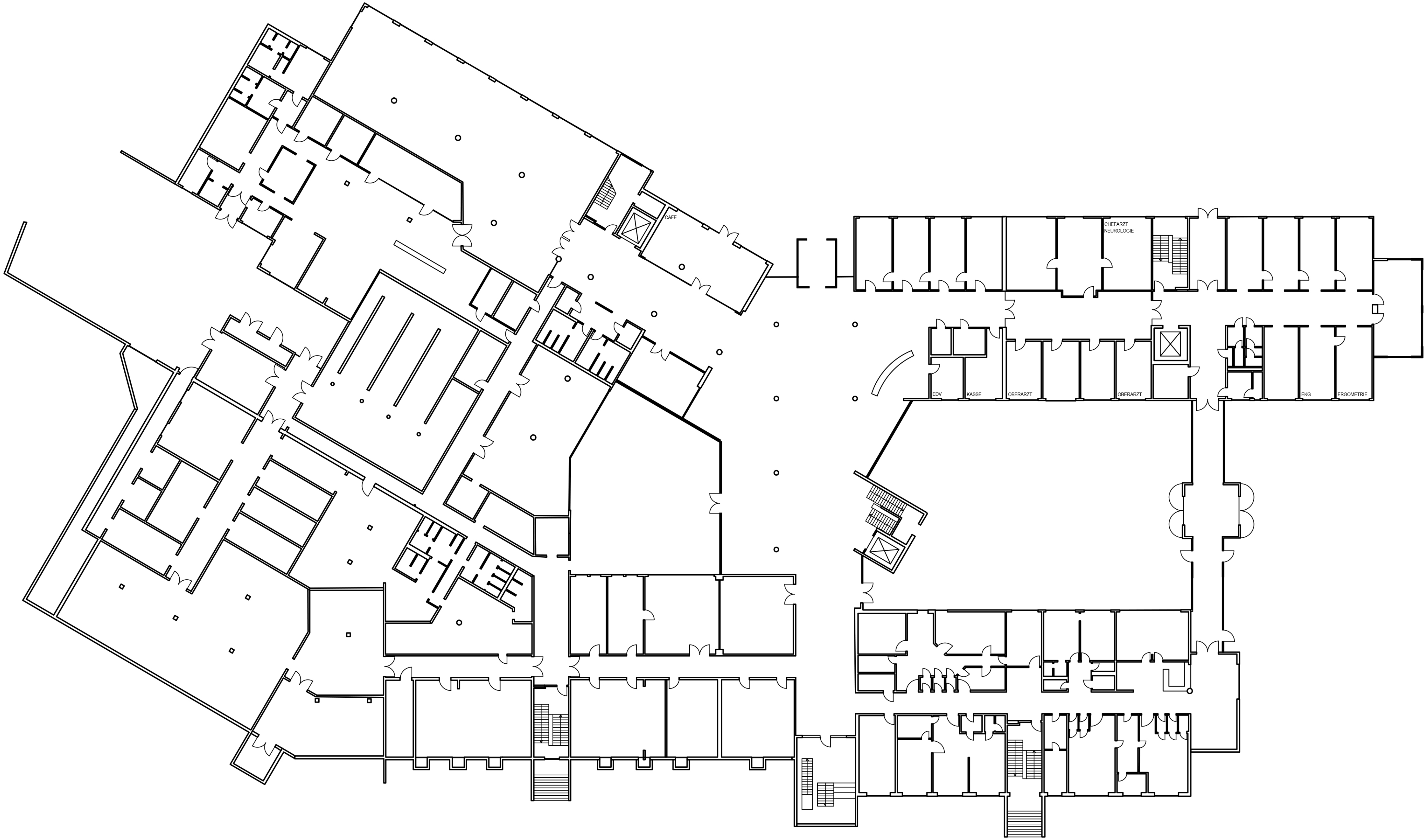

Symbols:

X - starting position, ———— - movement, O - stop, 1, 2, 3 - change of space, E - end position,

☐ - change of floor, P - patient, V - visitor, S - medical staff

Observation sheet no. ____________ Date _______________

Patient no. ____________________

1OG

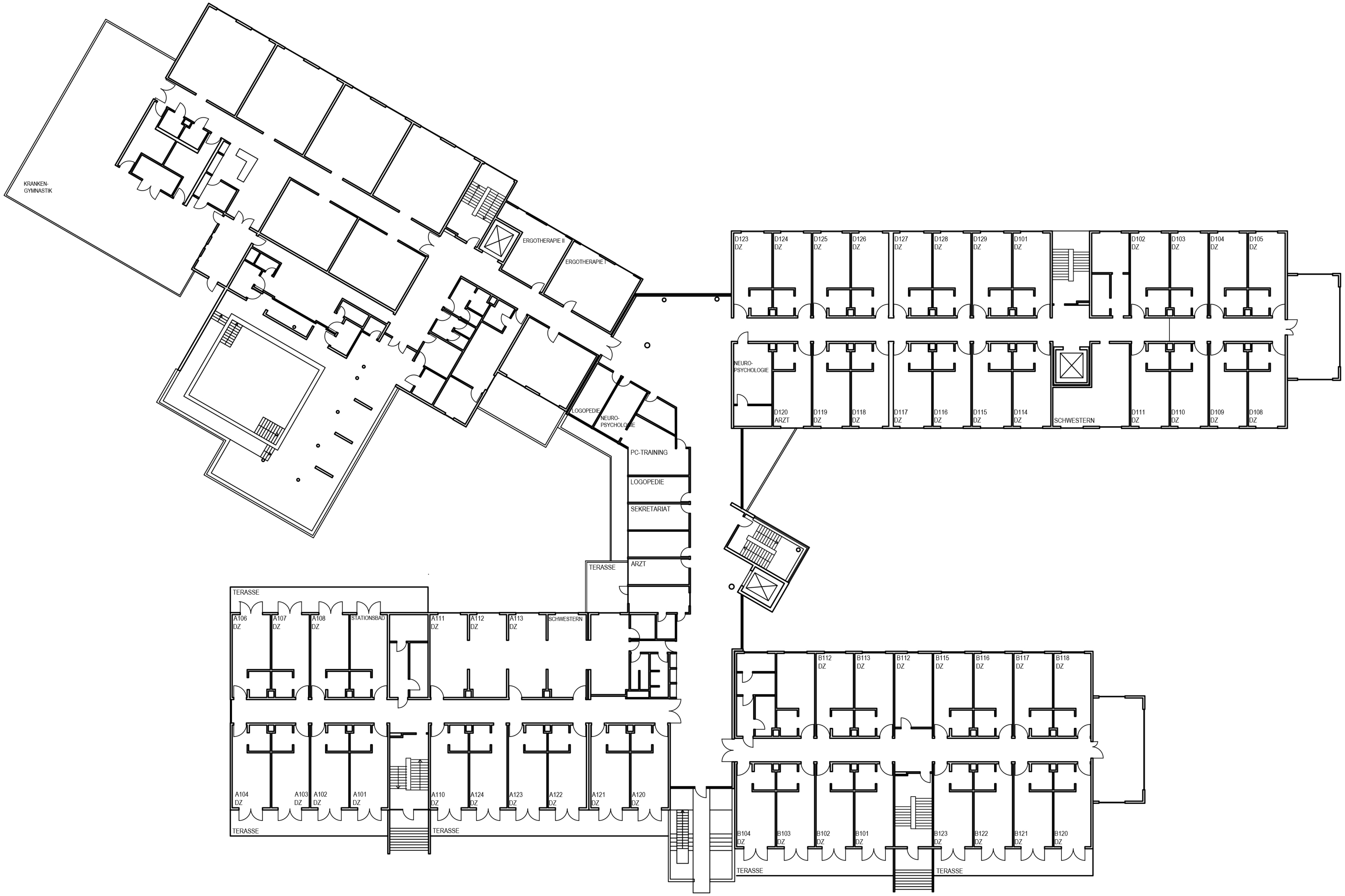

Symbols:

X - starting position, ——— - movement, O - stop, 1, 2, 3 - change of space, E - end position,

☐ - change of floor, P - patient, V - visitor, S - medical staff

Observation sheet no. ____________ Date _______________

Patient no. ____________________

2OG

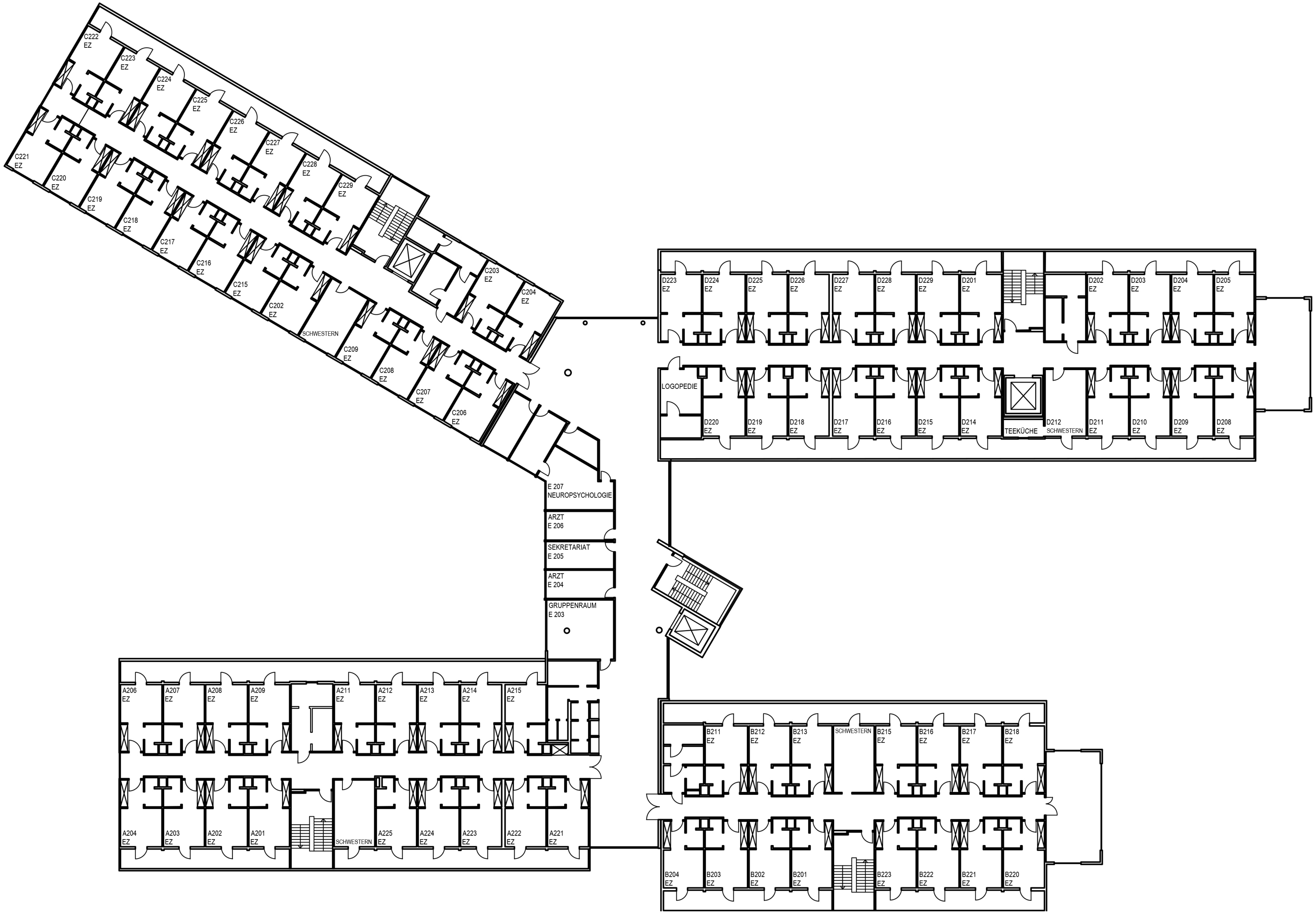

Symbols:

X - starting position, ——— - movement, O - stop, 1, 2, 3 - change of space, E - end position,

☐ - change of floor, P - patient, V - visitor, S - medical staff

Observation sheet no. ____________ Date _______________

Patient no. ____________________

3OG

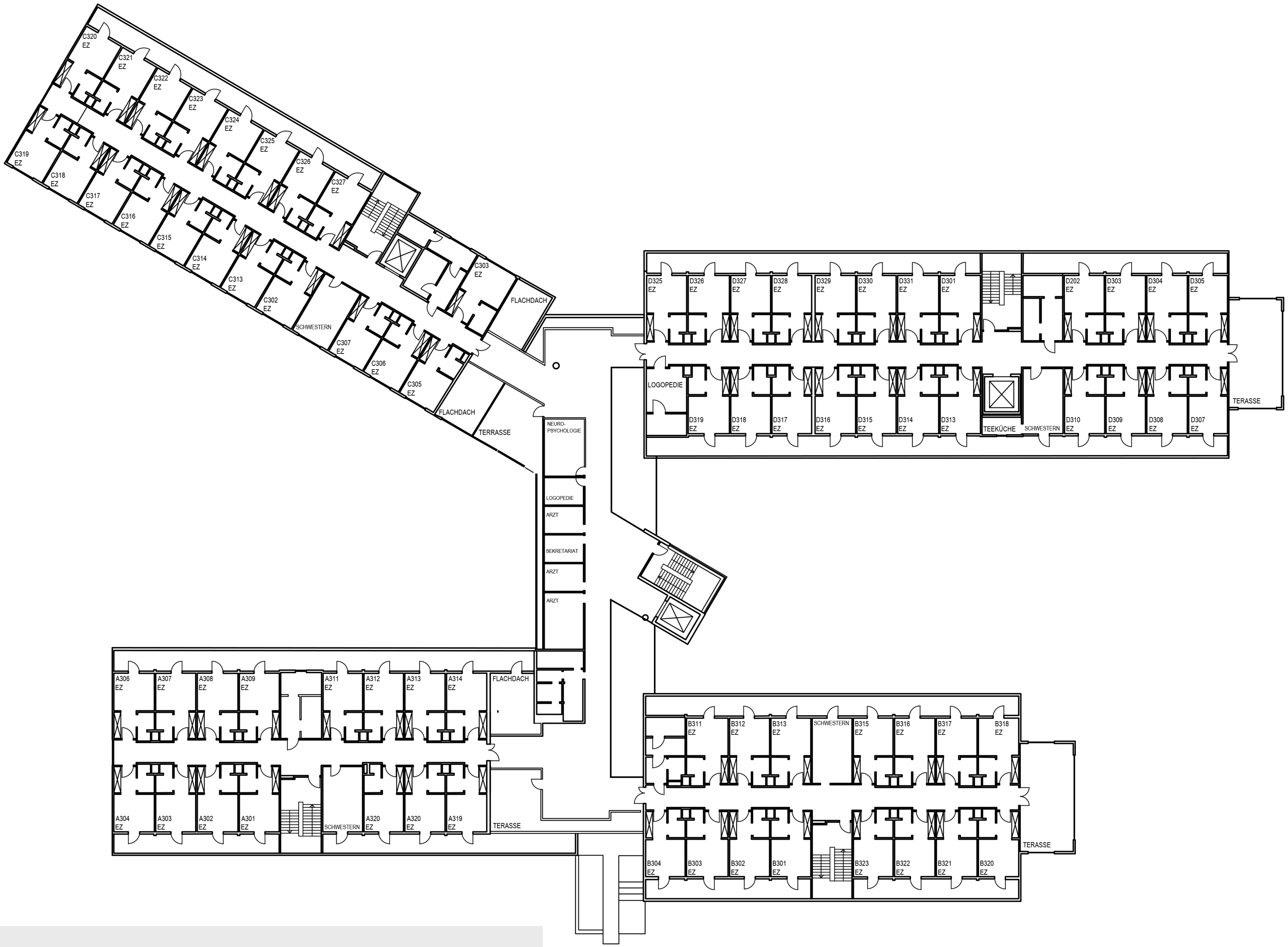

Symbols:

X - starting position, ——— - movement, O - stop, 1, 2, 3 - change of space, E - end position,

☐ - change of floor, P - patient, V - visitor, S - medical staff
