# Supplementary material for: Stroke Patients’ Free-Time Activities and Spatial Preferences During Inpatient Recovery in Rehabilitation Centers
Source: HERD. 2022 Jul 18;15(4):96–113. doi: 10.1177/19375867221113054 (PMC9523820; doi:10.1177/19375867221113054)
Supplement: Supplemental Material, sj-pdf-2-her-10.1177_19375867221113054 - Stroke Patients’ Free-Time Activities and Spatial Preferences During Inpatient Recovery in Rehabilitation Centers [file sj-pdf-2-her-10.1177_19375867221113054.pdf]

Supplemental Figure: Characteristics of the participating rehabilitation centers

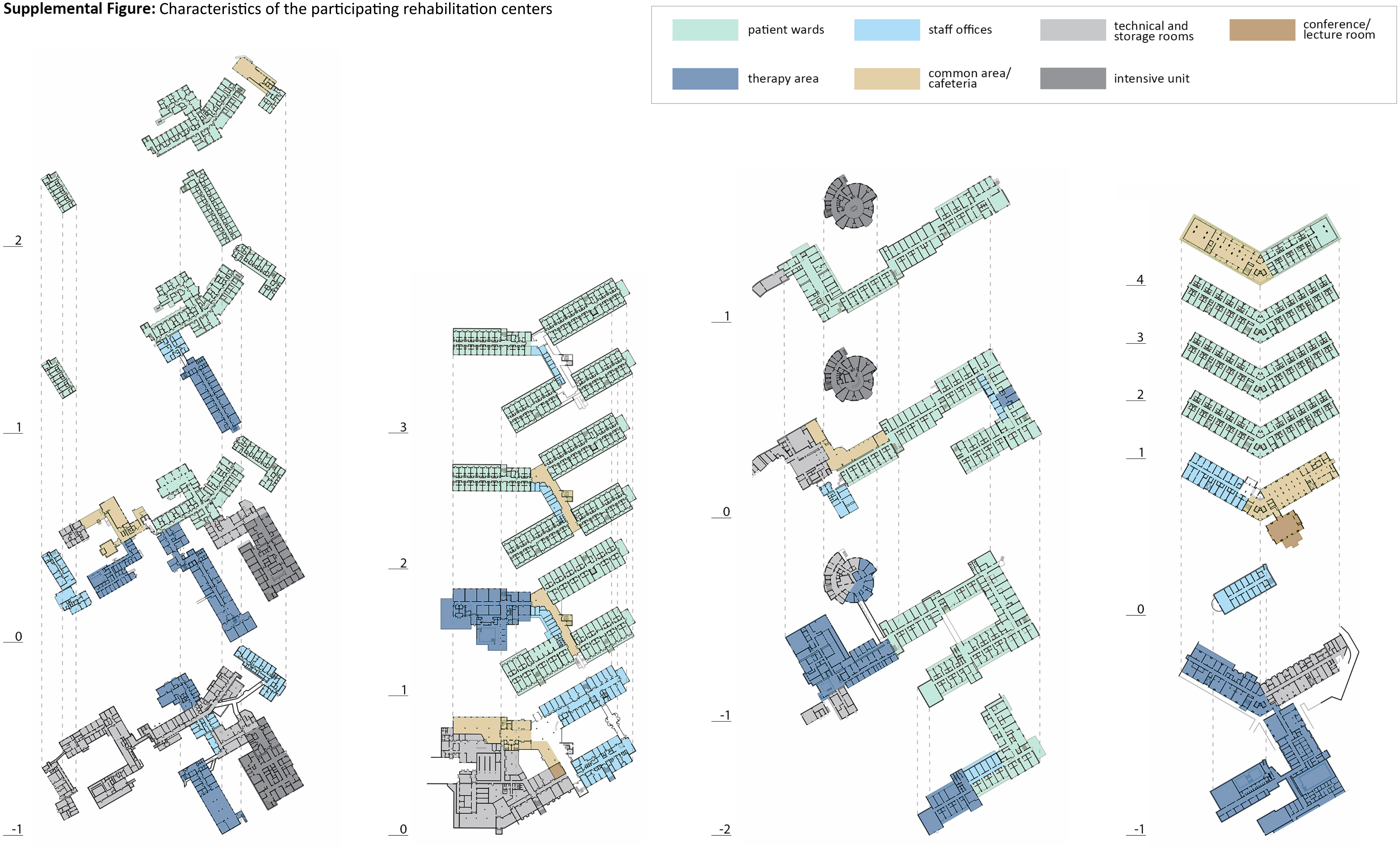

Center A

Number of beds: 240
Total surface: 19702.7 m^2
Typical floor surface: 3829.2 m^2

Center B

Number of beds: 210
Total surface: 18024.7 m^2
Typical floor surface: 3539.1 m^2

Center C

Number of beds: 225
Total surface: 17505.2 m^2
Typical floor surface: 5512.4 m^2

Center D

Number of beds: 188
Total surface: 13056.4 m^2
Typical floor surface: 1660.6 m^2

Supplemental Figure: Characteristics of the participating rehabilitation centers

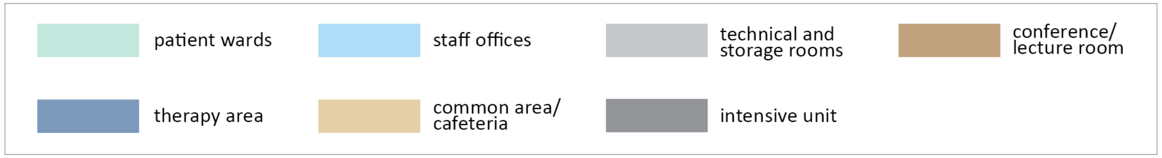

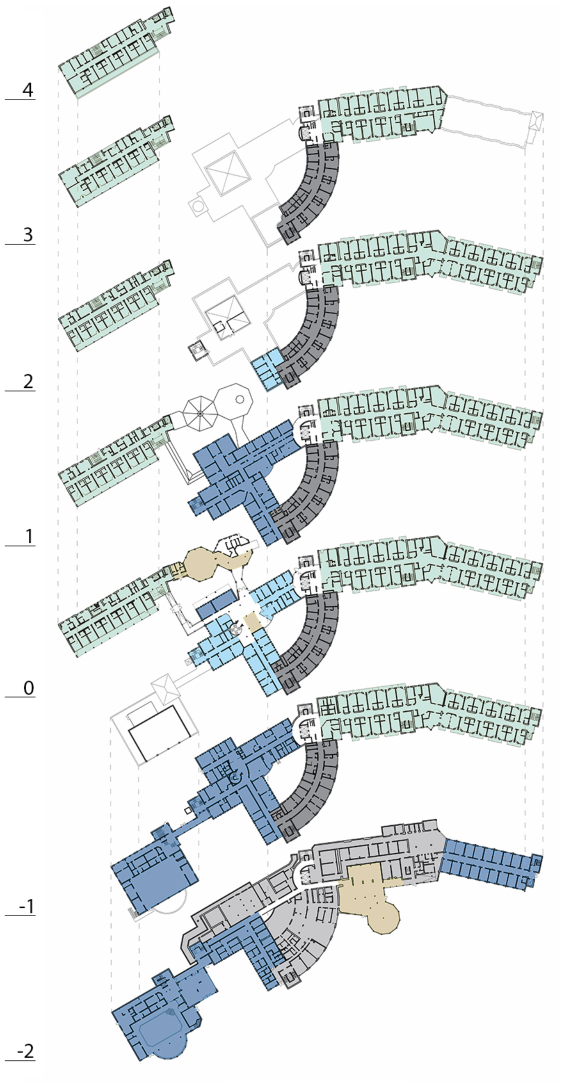

Center E

Number of beds: 250
Total surface: 22730.8 m^2
Typical floor surface: 4024.6 m^2

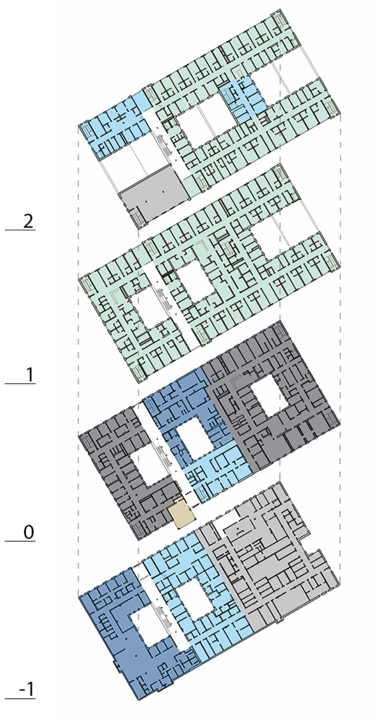

Center F

Number of beds: 210
Total surface: 18024.7 m^2
Typical floor surface: 3539.1 m^2

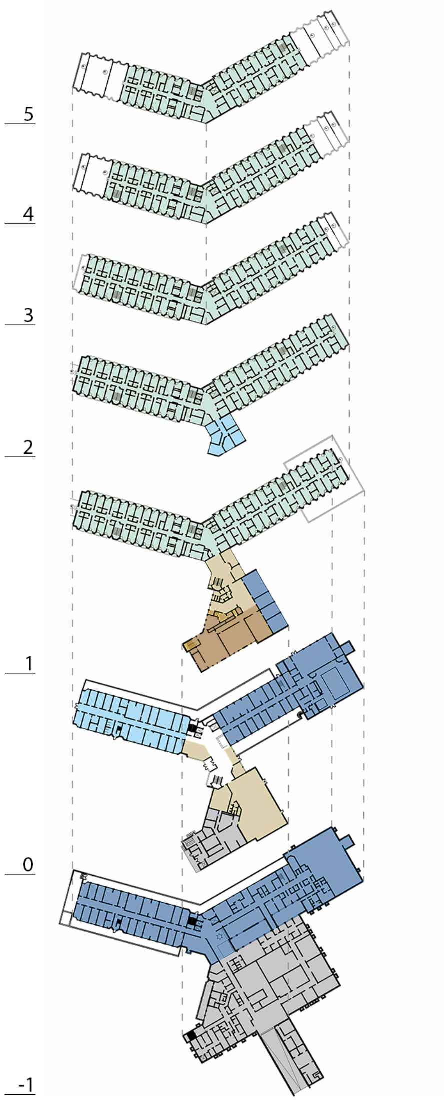

Center G

Number of beds: 225
Total surface: 17505.2 m^2
Typical floor surface: 5512.4 m^2
