# Supplementary material for: Stroke Patients’ Free-Time Activities and Spatial Preferences During Inpatient Recovery in Rehabilitation Centers
Source: HERD. 2022 Jul 18;15(4):96–113. doi: 10.1177/19375867221113054 (PMC9523820; doi:10.1177/19375867221113054)
Supplement: Supplemental Material, sj-pdf-3-her-10.1177_19375867221113054 - Stroke Patients’ Free-Time Activities and Spatial Preferences During Inpatient Recovery in Rehabilitation Centers [file sj-pdf-3-her-10.1177_19375867221113054.pdf]

Supplemental Figure: Common rooms/spaces on the wards

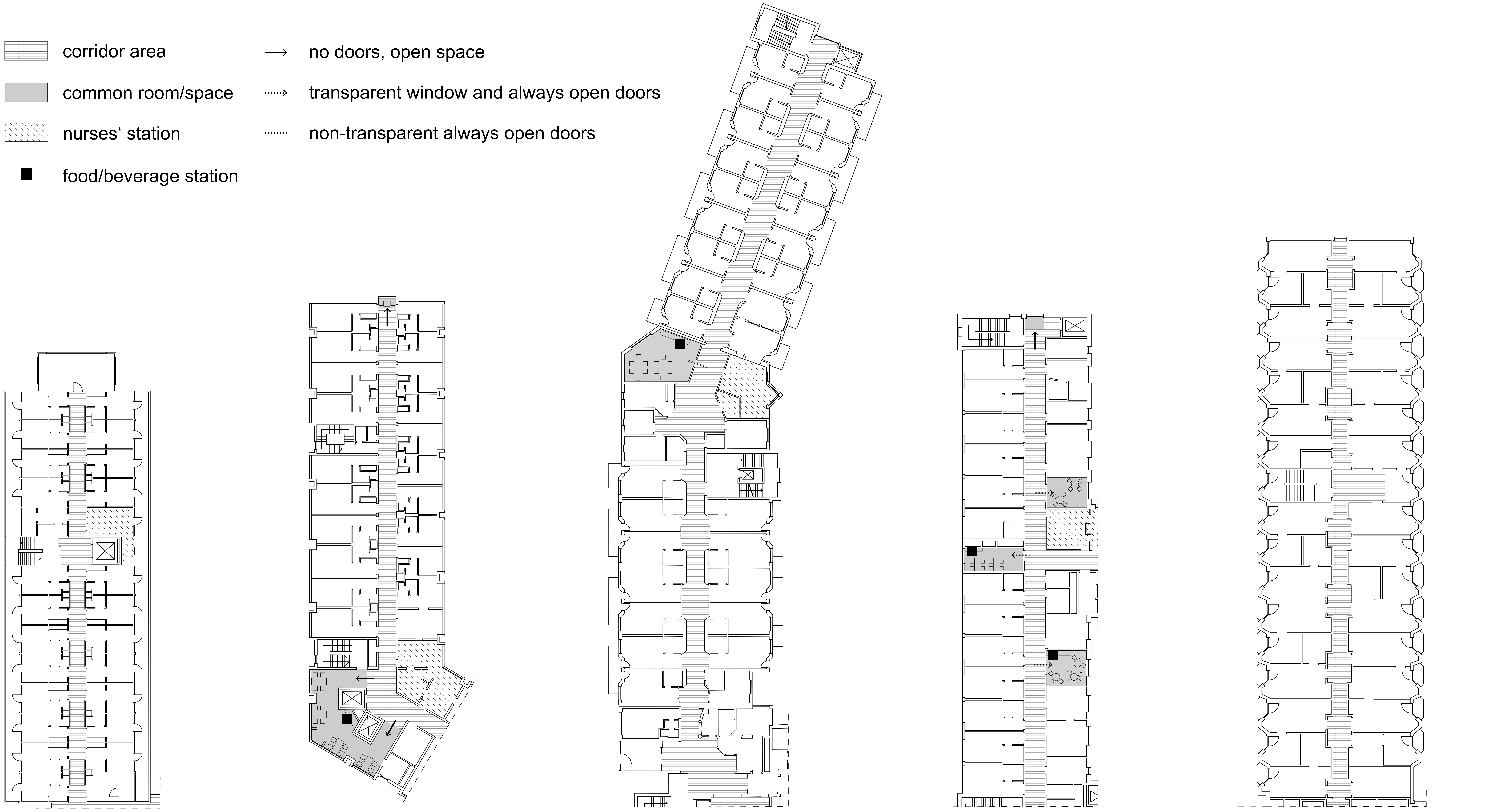

CENTER B

no common rooms on the wards

CENTER D

one common room per floor shared by two wards and a small corridor seating area

CENTER E

one main living/dining room on the ward

CENTER F

multiple living/dining rooms on the ward and a small corridor seating area

CENTER G

no common rooms on the wards, nurses‘ station between two wards
