# Supplementary material for: Stroke Patients’ Free-Time Activities and Spatial Preferences During Inpatient Recovery in Rehabilitation Centers
Source: HERD. 2022 Jul 18;15(4):96–113. doi: 10.1177/19375867221113054 (PMC9523820; doi:10.1177/19375867221113054)
Supplement: Supplemental Material, sj-pdf-4-her-10.1177_19375867221113054 - Stroke Patients’ Free-Time Activities and Spatial Preferences During Inpatient Recovery in Rehabilitation Centers [file sj-pdf-4-her-10.1177_19375867221113054.pdf]

Supplemental Table: Patients' responses to Q6 (n = 26)

Patients' responses	Categories	Themes
Cafeteria - to receive visitors, to meet with other patients.	Space to socialize with visitors and patients	Wanting to socialize
A room where you can sit with several people to read, talk, play. Close to my room on the same floor.		
Café or bistro open until 22:00h for socializing.	Space to socialize that is open longer in the evening	
A space where you can play board games.	Space for activities related to entertainment	Variety of common spaces is desired to support different activities
Music room, craft room		
Cinema room, dancing room, singing room		
Game room (for chess, etc.)		
TV room, sitting room		
A common area on my ward to sit, read.	Space to read/retreat	
Smaller room where you can retreat.		
A relax room. Many sun-loungers in a botanical garden. Like indoor Central Park…	Space to relax	
A pleasant common room.	Pleasant atmosphere, comfortable furniture, non-clinical style	Atmosphere, comfort, style, view are important
Room with appropriate furnishing.		
Living room on the ward, comfortably furnished.		
A comfortable sitting room, homely furnished, not in the hospital style, but as a café.		
With a nice view or a balcony in front.	Nice view	
Bright and quiet space	Quiet space, not many people	
Larger common rooms. I do not like rooms with many people.		
A common room that is easy to reach. The common room here in the clinic is far away. The room is very unpleasant and it is not inviting to stay.	Space that is easy to reach	
Many sitting areas, cafeteria, a smoking room for winter would be nice.	Cafeteria, sitting areas	Other
Cafeteria		
Cafeteria, terrace		
Cafeteria, balcony, terrace		
Various sitting areas		
Swimming pool	Other	
Library		
