# Supplementary material for: Stroke Patients’ Free-Time Activities and Spatial Preferences During Inpatient Recovery in Rehabilitation Centers
Source: HERD. 2022 Jul 18;15(4):96–113. doi: 10.1177/19375867221113054 (PMC9523820; doi:10.1177/19375867221113054)

Supplemental Figure: Paths of all observed patients in center B aggregated

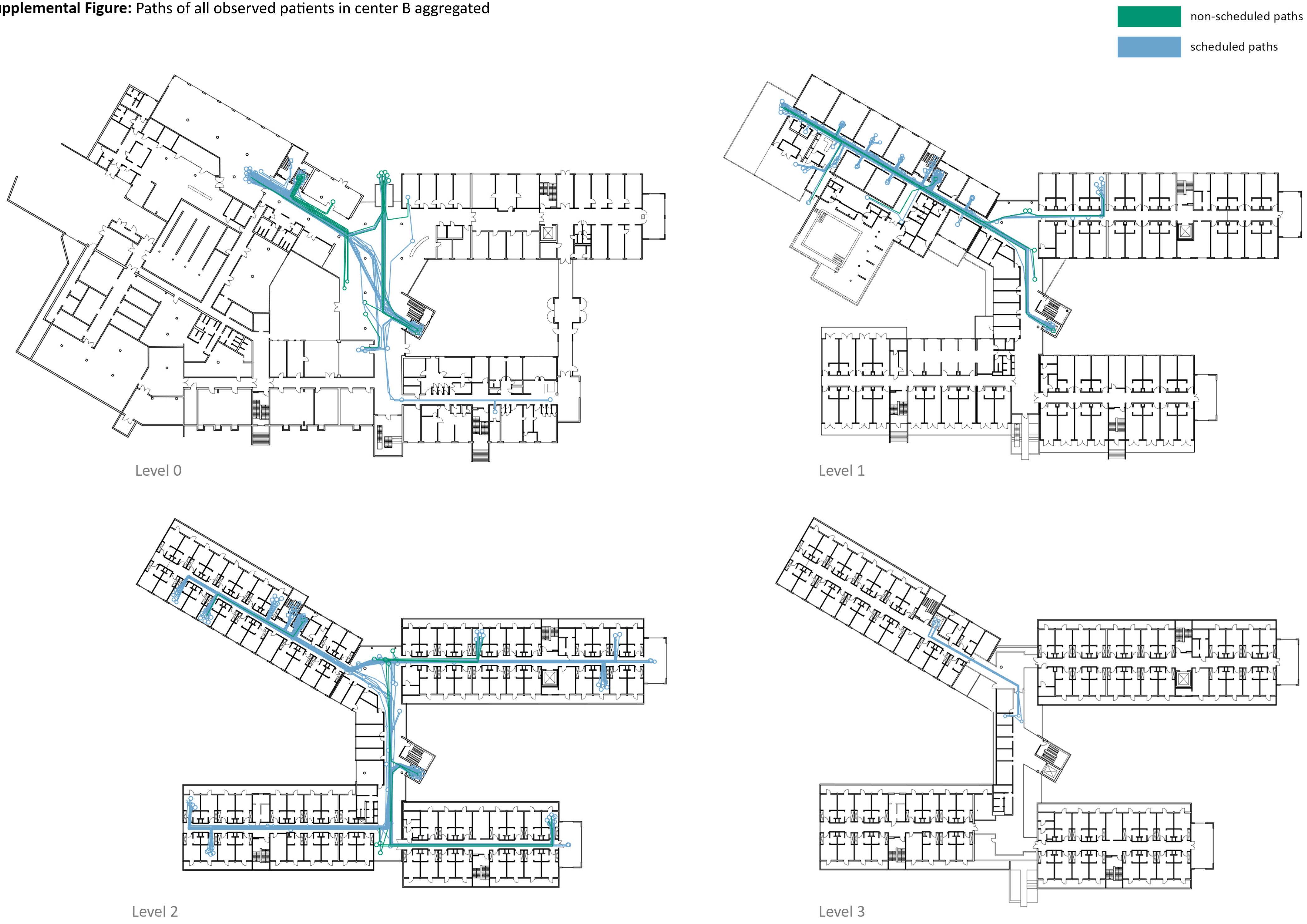

Supplement: Supplemental Material, sj-pdf-5-her-10.1177_19375867221113054 - Stroke Patients’ Free-Time Activities and Spatial Preferences During Inpatient Recovery in Rehabilitation Centers [file sj-pdf-5-her-10.1177_19375867221113054.pdf]
